# Do they really coexist? An empirical analysis of a conjoint implementation of Quality Management System and High Performance Work System on organizational effectiveness

**DOI:** 10.1371/journal.pone.0229508

**Published:** 2020-03-09

**Authors:** Khurram Rehmani, Yasir Ahmad, Afshan Naseem, Tasweer Hussain Syed

**Affiliations:** Department of Engineering Management, College of Electrical and Mechanical Engineering, National University of Sciences & Technology (NUST), Islamabad, Pakistan; Sichuan University, CHINA

## Abstract

Over the past few decades, Quality Management System (QMS) and High Performance Work System (HPWS) have emerged as key concepts to enhance organizational effectiveness. All over the globe, majority of contemporary manufacturing and services organizations have applied at least one of these development strategies or even both. This study proposes an integrated framework of QMS and HPWS and empirically investigates the relationship between QMS and HPWS practices and their direct and indirect effects on organizational effectiveness using structural equation modelling (SEM). This research makes a number of significant contributions: (1) The black box of the conjoint implementation is opened up for better appreciation of the interplay of QMS and HPWS practices and their influence on organizational effectiveness (2) The key QMS practices recognized as contributing factor of performance have been classified and examined at two distinct levels i.e. QMS Top Level practices and QMS Core practices (3) The mediating and interaction effects of QMS Core practices and HPWS practices on the relationship of QMS-Top Level practices and organizational effectiveness have been thoroughly investigated. The proposed framework is tested through cross-sectional data from 90 Technical Services Organizations (TSO) operating in Pakistan. The research hypotheses are supported by the test results of the SEM. The findings and implications are discussed along with limitations and future research guidelines.

## 1. Introduction

In an ever growing competitive environment, few would advocate an organizational philosophy that admits ‘we are good enough’. The consequences of such belief might result into low quality products, delayed schedules, increased complaints, and deteriorating satisfaction scores. Operationally, failure of an organization to produce products and services as per customer’s expectations has a significant cost. For any organization to survive and remain effective, the need for improvement in quality standards and enhancement in organizational effectiveness is ever present. The evolution of operation management practices such as QMS and latest trends in HPWS with their significant effect on organizational effectiveness, has received widespread attention from both academia and industry during the past few decades. Different frameworks of QMS [1, 2, 3, 4, 5] and HPWS [6, 7, 8] have been developed in the past to investigate their link with organizational effectiveness. In most of the QMS related studies, while analysing the relationship between QMS and organizational performance: (1) QMS has either been operationalized as a *single construct* [9, 10, 11] etc., or a *multidimensional construct* [12, 13, 14, 15] (2) the mutual relationship of quality management common practices and their impact on quality related outcomes have been focused [1, 2]. However, within the QMS, there is shortage of empirical evidence about the relationship between QMSTop Management practices and QMSCore practices, when both are operationalized as separate multidimensional constructs. Moreover, another neglected aspect is the fact that synergy and congruence within the QMS practices also have a significant impact on HPWS e.g. as asserted by Wilkinson [16] and Escrig-Tena et al. [17] QMS comprises of two different sides, one is “hard” and the other is “soft” and the “soft” side focuses on HRM. Different researchers have proposed some frameworks by combining QMS and HPWS such as Bayo-Moriones et al. [18] and Jimenez-Jimenez and Martınez-Costa [19] have analysed the effect of QM practices on HRM; however both the studies fell short to incorporate the basic ingredient of HPWS i.e. the concept of Human Resource (HR) bundle and relied upon individual HR practices only. Some other authors like M. de Menezes et al. [20], Obeidat et al. [21] and Alkhazali et al. [22] also explored the relationship between QMS and HPWS but the potential impact of integrated practices of QMS and HPWS on organizational effectiveness remained unanswered. Few researchers have simply presented some theoretical frameworks of QMS and HPWS while considering QMS as a single-level construct and HPWS as a group of individual HR practices [23, 24]. The objective of this study is to develop an integrated framework of QMS and HPWS such that: (1) the relationship within QMS practices can be investigated at two distinct levels i.e. Top Management level QMS practices and Core level QMS practices (2) the relationship between QMS and HPWS practices can be examined and (3) the effect of conjoint implementation of these two management systems on organizational effectiveness can be empirically analysed. The remaining paper is structured as follows. In Section 2, literature review of related studies is carried out to study the relationship of QMS, HPWS and organizational effectiveness and the outcomes of previous studies are summarized. In Section 3, the theoretical framework and research model along with the corresponding hypotheses is developed. Section 4 presents the research methodology that includes data collection processes, development of instrument and test of reliability and validity. Section 5 depicts the test results of structural model. Section 6 comprises of results discussion and implications, suggestion for future research and limitations of current study followed by conclusion.

## 2. Literature review

### 2.1 QMS and organizational effectiveness

QMS is a systematic approach consisting of mutually supporting principles, backed by multiple practices, criteria, tools and techniques, to achieve and sustain improved and high quality output [25]. There are varieties of definition for QMS in management literature based on following common aspects: (1) accomplishment of requirements as per specific customer needs (2) emphasis on particular products and/or services (3) enhanced organizational effectiveness and (4) lack of faults [26, 4, 13]. QMS has evolved as a management paradigm and an integrative management philosophy derived from multiple sources with several titles and acronyms. Likewise, the QMS theory has evolved from three different sources: (1) fundamental theories and concepts developed by quality gurus like Deming [27] and Juran [28] (2) quality award models such as European Quality Award (EQA), Malcolm Baldrige National Quality Award—MBNQA, Deming Award and (3) measurement studies carried out by researchers like Saraph et al. [29], Flynn et al. [25], Powel, [30], Ahire et al. [31], Dow et al. [32], Kaynak [1] etc. In the evolution process of this philosophy, several QMS practices have been identified, documented and empirically tested in various studies. However, the nature of analytical frameworks developed to probe the relationship between QMS and performance varies among the measurement studies. More recently, researchers such as Augustyn et al. [12], Abbas [13], Jimoh et al. [14], Sila [33], Shafiq et al. [34], and O’Neill et al. [4] have operationalized QMS as a ‘*Multidimensional Second Order Construct’* to analyse the relationship between QMS and organizational effectiveness, while other researchers Busu [9], Androwis et al. [10], Ahmad et al. [35] and Wokabi [36] have tested the effect of QMS on organizational effectiveness using a ‘*First Order Construct*’ consisting of several factors representing groups of QM practices. Some authors have also classified QMS practices into distinct groups e.g. the analysis of 145 studies by Hietschold [26] reveals that critical success factors of QMS can be classified as management tasks, core tasks and supporting tasks.

### 2.2 HPWS and organizational effectiveness

The term HPWS refers to a system of integrated or combined HR practices, work structures, and processes, designed to produce high levels of employee knowledge, skill, attitude, motivation and flexibility [37, 38]. A vast number of research studies support and prefer the concept of “*bundles*”, “*systems*” or “*configurations*” of HR practices over individual HR practices [8, 7]. The main argument given to support this concept is: individual HR practices perform well when applied in a combination instead of isolation with each other as employees are subjected to multiple tasks at the same time. An effective strategy in HRM must constitute three fundamental components: Abilities, Motivation and Opportunities [39]. Employee’s abilities are generally linked with effective recruitment and selection process coupled with extensive training programs [40]. Employee’s motivation and commitment refers to the fact that employees require necessary motivation to display discretionary efforts. This factor is typically linked with practices like employee’s compensation and incentives, performance management, and job security [40]. Finally, employee’s opportunity to contribute in shop-floor decisions includes practices related to information sharing, employee’s autonomy and team work [41]. Several researchers have established a significant positive relationship between HPWS practices and organizational effectiveness [6, 7, 37, 42, 41, 43]

### 2.3 Link between QMS and HPWS

As stated by various researchers and practitioners, collaboration and synergy among HPWS practices are essential for successful implementation of QMS; similarly, the failure is mostly because of poor HRM [18, 44]. Therefore, organizations are required to align their quality-oriented HRM system with quality objectives/goals for better organizational outcomes [44]. This argument has been supported earlier by various studies e.g. Powell [30] argued that the success of QMS is affected more by implicit and behavioural resources like cordial organizational culture and empowered/committed workforce than other technical elements. Dow et al. [32] established a positive relation of workforce commitment, collective vision, and customer satisfaction with quality of product. Samson and Terziovski [15] asserted that QMS is largely influenced by top management commitment, HRM and customers focus. Similarly, Caudron [45] stated that in most of the Baldrige Award winning organizations, HRM department is effectively integrated with other departments to support a successful QMS implementation.

The relationship of QMS and HPWS with organizational effectiveness identified in various studies is illustrated in “Table 1”.

**Table 1:** Relationship of QMS and HPWS with organizational effectiveness.

**QMS and Organizational Effectiveness**				
**Study**	**QM Practices**	**Performance measures**	**Data collection technique and type of analysis**	**Key Findings of study**
Samson and Terziovski [15]	Management leadership Employees Commitment Personnel Training Team Work Use of Benchmarking Use of JIT Customer Focus Shared Vision	Product Quality, Customer Satisfaction, Productivity, Delivery Performance	Questionnaire/ Structural Equation Modeling, Multiple Regression Analysis	The combination of employee commitment, shared vision, and customer focus is positively related to quality outcomes. Whereas, there is a positive link between management leadership, HRM practices and customer focus with firm’s operational performance
Ho et al. [46]	Core and Supportive QMS constructs	Product Quality	Questionnaire/ Hierarchical Regression Analysis	The relationship between QMS supportive practices and product quality is mediated through QMS core practices
Prajogo and Sohal [47]	Leadership Strategic Planning Customer Focus Process Management Information and Analysis People Management	Quality Performance, Innovation Performance	Questionnaire/ Regression Analysis	There is a positive relationship between the QMS-mechanistic elements and quality performance and between QMS- organic elements and innovation performance.
Tari et al. [2]	Leadership Quality Planning Process Management HRM Continuous Improvement Supplier Management	Quality Performance	Path Analysis	The findings support the proposed relationships within QMS practices and a positive relationship also exists between these practices and quality outcomes.
Sadikoglu and Zehir [3]	Leadership Process Management Information and Analysis Supplier M|anagement Customer Focus Continuous Improvement	Customer Satisfaction, Product quality, Lead time	Questionnaire/ Structural Equation Modeling	A positive relationship is found between QMS and organizational performance which is also mediated through employee performance and innovation performance.
García-Bernal and Ramírez-Alesón [48]	Level of QM implementation Level of intensity of QM used	Operational Performance, Customer Satisfaction	Questionnaire/ Structural Equation Modeling	A positive relationship is found between QMS and operational performance which further effects financial performance and customer satisfaction.
O’Neill et al. [4]	Employment Conditions Union Membership Quality Programs Business Intentions	Financial Performance	Longitudinal data/CAPLAB and VADLAB ratios	SAMFs who adopt QMS have better financial results than those who do not adopt this program.
Shafiq et al. [34]	Leadership Strategy People Partnership and Resources	Product Quality, Customer Satisfaction, Productivity	Questionnaire/ Structural Equation Modeling	QMS is positively linked with organizational effectiveness.
Escrig-Tena et al. [17]	QMS Hard Practices QMS Soft Practices	Product Innovation and Process Innovation	Questionnaire/ Structural Equation Modeling	Both hard and soft practices of QMS are positively linked with innovation performance, whereas proactive behaviour acts as a mediator between QMS hard/soft practices and innovation performance.
Abbas [13]	Leadership Strategic Planning HR Management Process Management Customer Focus Information Analysis	Environmental /Social/Corporate Sustainability	Questionnaire/ Structural Equation Modeling	QMS is positively linked with Corporate Sustainability. Knowledge Management Partially mediates QMS and CS relationship.
**HPWS and Organizational Effectiveness**				
**Study**	**HPWS Practices**	**Performance measures**	**Data collection technique and type of analysis**	**Key Findings**
MacDuffie [49]	Team based work system Contingent Compensation and Extensive Training etc.	Productivity and Quality	Questionnaire/ Regression Analysis	The systematic integration of HR bundles and manufacturing policies under a flexible production environment enhances both productivity and quality
Koch [50]	HR Planning Employees Development Employees Selection	Labour Productivity	Questionnaire/ Regression Analysis	Firms with a streamlined HR Planning and systematic recruitment and selection process have a positive impact on labour productivity
Ramsay et al. [51]	Problem Solving Groups Employees Consultation Incentives Communication Others	Product Quality Labour Productivity Financial Performance	Questionnaire/ Regression Analysis	Models based on HPWS and Labour process approaches are tested and are found wanting.
Subramony [52]	Employees Knowledge, skills and Abilities Employees Opportunities to Contribute Employees Motivation and Commitment	Various	Meta-Analysis	A meta-analysis of 65 research studies reveals that there is a greater magnitudes of impact of HRM bundles on business performance as compared to constituent individual practices
Bello-Pintado [53]	Ability Enhancing Bundles Motivation Enhancing Bundles Opportunities Enhancing Bundles	Efficiency Delivery Speed % of defective finished products	Questionnaire/ Regression Analysis	The HRM practices once applied in bundles have a significant positive effect on manufacturing performance
Shin and Konrad [7]	Training Empowerment Compensation	Competitive advantage/Productivity	Longitudinal data/ Structural Equation Modelling	HPWS has a significant positive effects on productivity /performance where performance also generates feedback in the form of information
Pak and Kim [6]	Team Manager’s implementation of Espoused HR practices	Employee /Team Performance	Survey/ Structural Equation Modelling	Team Managers have a significant relation with HPWS intensity which in turn enhances team performance

## 3. Theoretical framework and proposed hypothesis

Various practices of QMS and HPWS have extensively been discussed with reference to organizational effectiveness in management literature. The present study aims to empirically examine the conjoint implementation of these two management systems within an integrated framework. The practices referred to in this study have been identified from an extensive literature review. The known practices (critical success factors) of QMS have been categorized and examined at two distinct levels:

1. QMS Top Management practices:—Top Management Commitment, Strategic Vision and Planning and Quality Culture.
2. QMS Core practices:—Process Management, Customer Focus, Quality Assurance and Control and Continuous Improvement.

**Fig 1:**
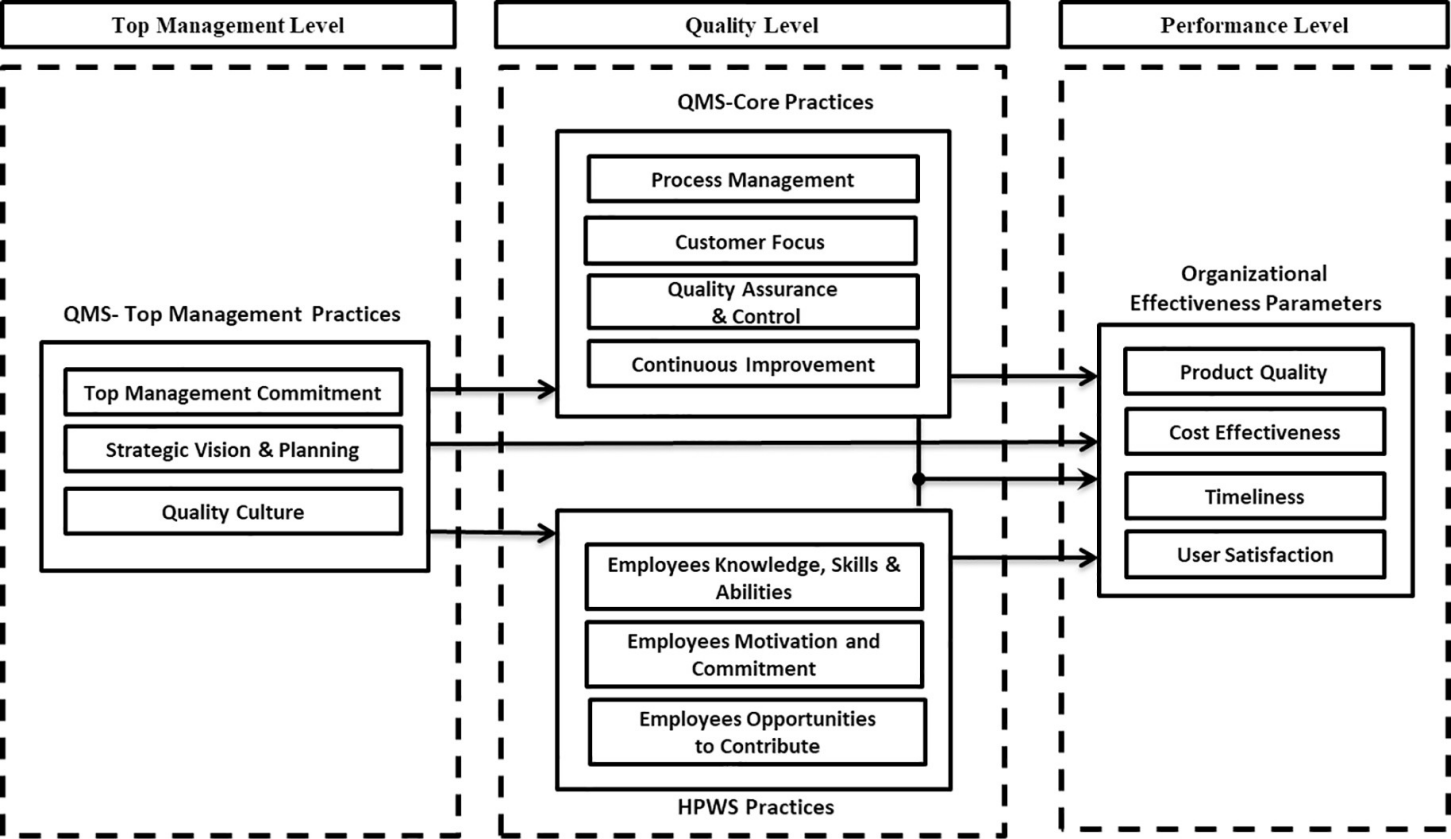
Theoretical framework of relationship between QMS, HPWS and OE parameters.

With respect to organizational effectiveness, aspects of timeliness, product quality, cost effectiveness and customer satisfaction are considered to provide insights on the effectiveness of the conjoint implementation of QMS and HPWS strategies. We hypothesize that TSOs can achieve significant improvement in terms of organizational effectiveness by implementing the proposed integrated framework of QMS and HPWS practices as shown below in Fig 1.

### 3.1 QMS-top level practices

Among the Critical Success Factors (CSFs) of QMS, few factors/strategic practices are considered as “*enablers”* or “*keys”* which drive other supporting/core practices [26]. These practices are recognized as Top Management practices. The initiation of other core quality activities stems from these top management tasks [26].

#### 3.1.1 Top Management Commitment

As recognized by top quality experts like Deming [27] and Juran [28], Top Management Commitment is one of the leading elements in QMS implementation as it enhances organizational outcomes by impelling other core QMS factors [54, 1]. Top Management Commitment sets the operational norms of QMS, clearly communicated along the entire organizational structure [55, 5, 56]. A committed management at the top plays a pivotal role by developing a clear organization mission and policy to achieve continuous improvement, communication, and collaboration amongst the value chain hence promoting a quality culture throughout the organization [57]. It is always challenging to bring an effective change in any organization without creating an environment where workforce is willing to accept the change and have fair opportunities to come out with the ideas, beneficial for the organization as a whole [58, 59].

#### 3.1.2 Strategic vision and planning

When viewed as a strategic imperative, QM shifts from being simply quality control or quality assurance and enters a strategic quality phase with heightened commitment to quality at all levels in the organization. Strategic vision and planning is the apex of the broader system of quality management that links performance, strategic objectives and competitiveness to quality efforts. The overall strategic planning must incorporate two key strategic business issues i.e. emphasis on customer focused quality and enhanced organizational performance [15]. Solis et al. [60] established a positive relationship between strategic quality planning and quality performance.

#### 3.1.3 Quality culture

Quality culture leads organizations to pursue functional excellence; it is viewing quality as an entire ‘system of thoughts’. A strong quality orientation at the top level helps recognition of “quality philosophy” throughout the organization [61]. Likewise, lack of quality awareness at the top level creates obstruction in problem-solving and hampers continuous improvement process in the organization [62]. Terziovski et al. [63] identified certain key elements linked with the establishment of a strong quality culture in organizations: integrated modifications in organizational system, quality-based mission and goals, spread of quality awareness throughout the organization, benchmarking, customer focus and employees well-being.

Based on the reviewed literature above, following hypotheses are formulated.

**H1.** There is a significant positive relationship between QMS- Top Management practices and organizational effectiveness.
**H2.** There is a significant positive relationship between QMS- Top Management practices and QMS- Core practices.
**H3.** There is a significant positive relationship between QMS- Top Management practices and HPWS practices.

### 3.2 QMS-Core practices

A substantial consensus exists regarding the core practices of quality management in management literature [64, 65]. QMS-Core practices focused in this study are: Process Management, Customer Focus, Quality Assurance and Control and Continuous Improvement.

#### 3.2.1 Process management

Refers to alignment of processes with the strategic goals/objectives of organization through a clear demarcation of ownership and responsibility [5]. It is primarily linked with an effective design and implementation of process architectures and process measurement systems [55]. Evans and Lindsay [66] described process management as *“An effective design and introduction of products by integrating the requirement of production*, *delivery and management of the supplier’s performance”*. Flynn et al. [25] established that with effective process management techniques, organizations can achieve a substantial decrease in rework and improve their production quality along with cost effectiveness and on time delivery. The findings of Ahire and Dreyfus [31] also indicate that product quality is positively affected by process management. To ensure reduction in process variations, different preventive and proactive measures are taken at production stages e.g. designing streamlined and steady production schedules and appropriate work distribution [1, 25].

#### 3.2.2 Customer focus

Is the extent of commitment of an organization to determine the current and future customer requirement/expectations, establish customer relationship and evaluate the level of customer satisfaction [56]. Thus processes need to be tuned in accordance with customer’s feedback so as to enhance the quality of products [25]. Several studies have established positive links of customer focus with product quality, customer satisfaction and on time delivery [6, 5, 13].

#### 3.2.3 Quality assurance and control

A visible and autonomous quality department having direct access to top management and effective coordination with other departments, significantly influence quality of product and organizational outcomes [29, 67]. Imparting conceptual and practical training to quality department’s staff helps them to adequately handle quality-related issues that eventually lead to quality improvement [2]. Use of quality tools and techniques enables quality assurance and control department to ascertain potential improvement areas, resulting in effective monitoring and control over quality variations [27].

#### 3.2.4 Continuous improvement

Refers to evolving processes to explore superior approaches during the process of transforming inputs into outputs in an endless improvement loop [68]. It is an on-going effort to refine inter-linked processes in the light of their efficiency and flexibility [69]. In order to have an effective group problem-solving and collaborated decision making, work processes must be revised and upgraded constantly [70] and process management techniques need to be applied effectively [71]. Organizations can improve their output and product quality through: reduction in variation, decrease in rework and wastage of materials, mechanical efforts and workforce [72].

Following hypotheses related to QMS-Core practices are proposed.

**H4.** There is a significant positive relationship between QMS-Core practices and organizational effectiveness.
**H5.** QMS- Core Practices mediate the relationship between QMS-Top Management practices and organizational effectiveness.

### 3.3 HPWS practices

In contemporary literature, core HPWS practices have generally been categorized as three different bundles/sets: (1) Employees Knowledge, Skills and Abilities (2) Employees Motivation and Commitment and (3) Employees Opportunities to Contribute [52, 38].

#### 3.3.1 Employees Knowledge, Skills and Abilities (KSA)

KSA bundles are combination of HRM practices related to selection and training with primary emphasis on enhancement of collective knowledge, abilities, and skills of the employees [39]. Various studies have established a positive relationship between human capital and organizational effectiveness [73, 39]. A synergistic combination of *selection procedures* and *training practices* provides a solid platform to build a skilful workforce by selecting employees having basic KSAs and training them well to achieve high levels of organizational performance [74, 39].

#### 3.3.2 Employees Motivation and Commitment (EMC)

EMC bundles help in directing efforts of employees toward the achievement of organizational objectives and provide them with the stimuli, required to engage in high levels of performance. These bundles comprise motivational practices like: effective performance appraisal system to evaluate individual and team performance, associating these assessments with a fair incentive and compensation systems and job security of employees [37]. Employees while working toward the accomplishment of specific goals are expected to exercise high standards of performance once they receive continuous feedback on their tasks/behaviour and are sufficiently rewarded for performance [37].

#### 3.3.3 Employees Opportunities to Contribute (EOC)

EOC bundles are planned to designate the decision-making powers and responsibilities down the hierarchy by using self-managing/autonomous groups and assisting participation of employees through a feedback process [75]. Employee’s empowerment practices positively affect job-related performance of individuals through improvement in their self-efficacy standards [44] and organizational performance by enhancing employee’s collective potency levels and sovereignty [76].

Hence following hypotheses related to HPWS are proposed.

**H6.** There is a significant positive relationship between HPWS practices and organizational effectiveness.
**H7.** HPWS practices mediate the relationship between QMS-Top Management Practices and organizational effectiveness.
**H8.** The interaction between HPWS practices and QMS-Core practices has a significant positive impact on organizational effectiveness.

**Fig 2:**
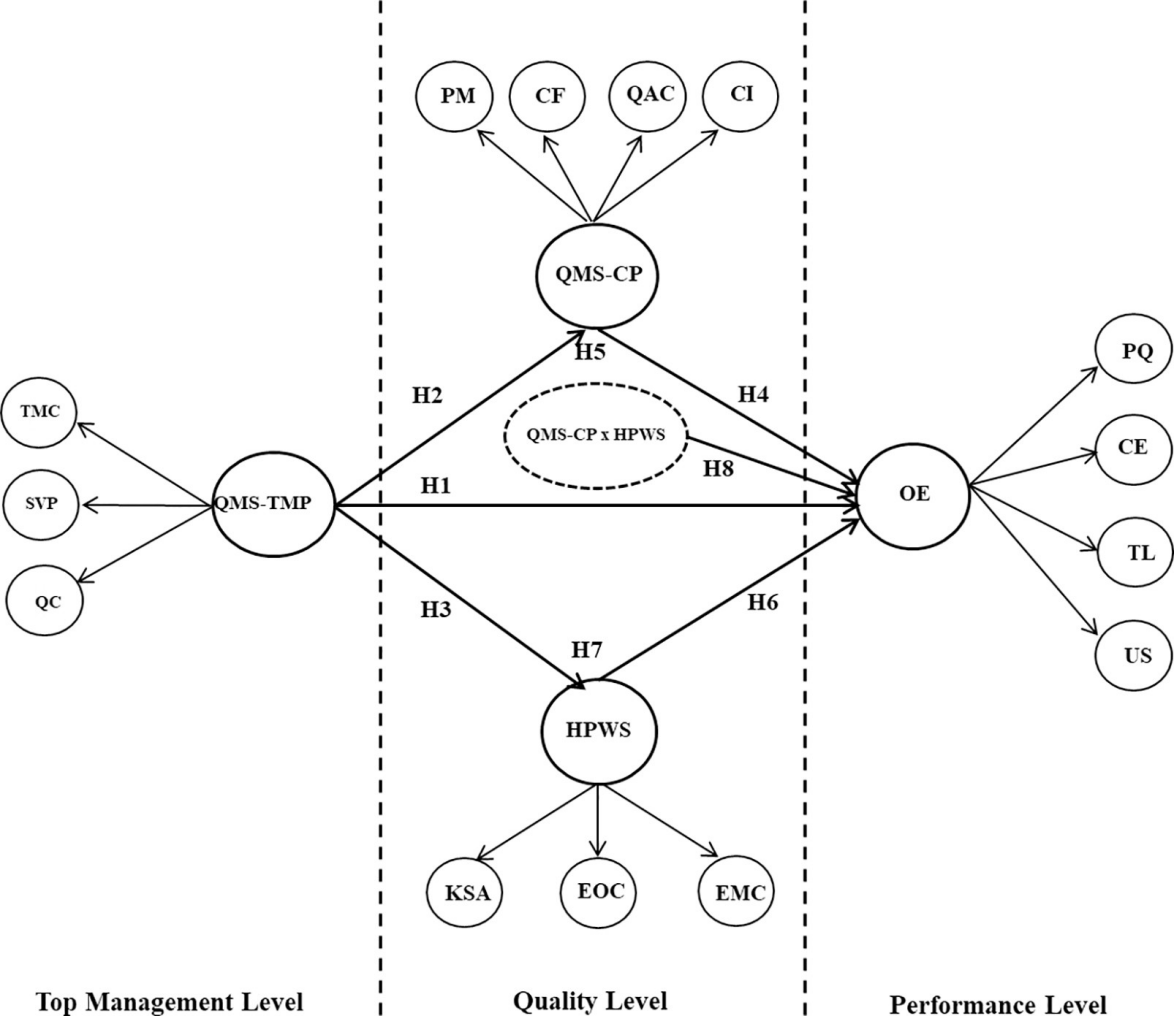
General structure of model with proposed hypothesis.

General structure of model with proposed hypothesis is presented in Fig 2 below.

## 4. Research methodology

### 4.1 Data collection

The data for this research study was collected through a cross-sectional mail survey of 90 TSOs of Pakistan after formal approval. Multiple volunteer respondents belonging to different hierarchies were engaged to have a transversal image of organization and avoid any key informant bias [77]. The respondents were fully informed about the objective of this study. Moreover, it was ensured that the data obtained was fully anonymized. A total number of 105 organizations were initially considered with 525 respondents (i.e. 5 respondents from each organization including CEO, Technical Managers, Quality Managers, Planning and Control Managers, Procurement Managers). It was not possible to acquire data from 7 organizations therefore remaining 98 organizations with 490 respondents were engaged. Finally 276 responses were received from 90 organizations (response rate: 56.32%) however 36 responses were eliminated due to incomplete data. The research is finally based on the data of 240 respondents. According to the size, 12 organizations are categorized as small organizations (<100 employees; 13%), 53 organizations as medium (100–250 employees; 59%) and 25 as large organizations (>250 employees; 28%). The collected data was checked for non-response bias by analysing the difference of the interest variables between early (63 organizations) and late respondents (17 organizations) [78]. Finally a sample t-test procedure was conducted for QMS, HPWS and four perceived factors of organizational effectiveness. None of the *t*-test outcomes showed any significant difference between the two response waves on the means of these variables.

### 4.2 Development of the instrument and measures

The questionnaire is developed in three subsequent stages. At first stage, pertinent literature is thoroughly reviewed to identify already developed measures with high degree of reliability and validity. Some items in the questionnaire are partially modified in accordance with the working environment of TSOs, however it is ensured that basic essence remains the same. At second stage, five TSOs experts are approached to offer their opinion on the suitability of our survey instrument. Their feedback is incorporated in the questionnaire. At third stage, the questionnaire is finally assessed by three prominent academic experts (PhDs) and five PhD scholars, subsequently some of the items are either eliminated or modified to establish content and face validity of the questionnaire [79]. The final version of questionnaire appears at S1 Appendix. To check Common Method Variance (CMV), Harman’s single-factor test is carried out and the results are further confirmed through Confirmatory Factor Analysis (CFA) using AMOS-20 with a Common Latent Factor (CLF). Both the results indicate that common method bias is not a serious concern in this study. The questionnaire comprises of three major parts. The first part is related to the respondent and common information of organization (demographics). The second part consists of independent variables, measured on seven-point Likert scales form “strongly disagree = 1, neutral = 4, strongly agree = 7”. Third part is about organizational effectiveness measures, respondents were requested to rate their organization as compared to their key competitors on seven-point Likert scales as “well below average = 1, neutral = 4, well above average = 7”.

### 4.3 Test for reliability and validity

This section reports the test results for reliability and three components of construct validity: unidimensionality, convergent validity and discriminant validity. Each scale of QMS, HPWS and organizational effectiveness constructs is assessed for reliability through calculation of Cronbach’s *α* [80]. Two items within the Top Management Commitment, three items within Process Management and one item within Employees Knowledge, Skills and Abilities are dropped due to low value of “*α”*. Rest all have adequate reliability level, value of *α* ≥ 0.70 [81]. To establish the unidimensionality of factors, Exploratory Factor Analysis (EFA) using “Principal Component Extraction with a varimax rotation” is performed [1]. All the communalities are higher than 0.7 and qualify the factor analysis criteria of 0.6 as recommended by MacCallum et al. [82]. EFA results are shown in Table 2. Scree plots and Eigen values shows that items are loaded on eleven explicitly identifiable factors having Eigen value more than “**1”** (minimum 1.162 to maximum 12.086) and factor loadings range from 0.74 to 0.92. The reliability of items is further confirmed through EFA results. Cumulative variance explained by all the factors is also sufficient (87.83%) [83].

**Table 2:** Exploratory factor analysis.

	Component										
	1	2	3	4	5	6	7	8	9	10	11
TMC1	.900	.188	.073	.056	.078	.059	.133	.074	.107	.043	.170
TMC2	.921	.155	.058	.055	.106	.010	.117	.100	.106	.065	.168
TMC3	.908	.194	.075	.040	.106	.034	.120	.098	.125	.024	.186
TMC4	.877	.217	.079	.059	.111	.002	.156	.063	.101	.016	.165
SVP1	.215	.285	.078	.014	.073	.116	.004	.098	.092	.028	.860
SVP2	.243	.277	.034	.029	.017	.082	-.008	.107	.067	.076	.880
SVP3	.288	.283	.058	.018	.027	.059	.009	.150	.130	.077	.814
CF1	.232	.850	.084	.068	.131	.078	.059	-.041	.104	-.005	.245
CF2	.186	.892	.090	.079	.109	.067	.053	-.013	.088	.034	.172
CF3	.180	.886	.060	.072	.061	.086	.076	.055	.180	.005	.166
CF4	.145	.904	.057	.101	.054	.043	.063	.059	.100	.008	.186
PM1	.040	.068	.199	.883	.198	-.026	.162	.009	.128	-.070	.018
PM2	.060	.069	.228	.901	.169	.008	.180	-.036	.127	-.009	.008
PM3	.052	.115	.239	.894	.186	.014	.184	.011	.101	-.013	-.015
PM4	.058	.082	.232	.829	.220	-.004	.205	.024	.078	.021	.057
IM1	.046	.064	.872	.260	.185	.008	.183	.004	.075	.019	.056
IM2	.051	.077	.917	.232	.164	-.027	.135	-.006	.039	.004	.009
IM3	.101	.067	.882	.194	.195	.009	.208	.016	.045	.067	.017
IM4	.097	.096	.844	.214	.214	-.004	.269	-.016	.051	-.024	.106
QAC1	.147	.019	.190	.209	.234	-.038	.838	-.009	.142	.009	-.015
QAC2	.122	.042	.160	.177	.234	.033	.851	-.001	.076	-.009	.018
QAC3	.153	.115	.199	.182	.117	.008	.865	-.091	.104	.069	.006
QAC4	.117	.080	.231	.166	.210	.030	.842	-.010	.078	.016	.000
CI1	.108	.088	.218	.187	.874	.043	.199	.033	.125	.024	.053
CI2	.109	.100	.213	.201	.878	.041	.210	.054	.123	.024	.048
CI3	.099	.123	.151	.216	.854	.016	.201	.055	.108	.009	-.016
CI4	.105	.065	.195	.189	.874	.031	.198	.102	.111	.045	.051
KSA1	.023	.078	-.044	.005	.038	.874	.003	.227	.097	.207	.061
KSA2	-.003	.061	.013	-.023	.055	.828	.038	.255	.164	.172	.076
KSA3	.044	.056	-.041	.005	-.001	.890	.016	.218	.103	.215	.038
KSA4	.033	.078	.058	.005	.021	.870	-.018	.201	.149	.164	.067
EOC1	.079	-.005	-.032	.005	.007	.275	-.074	.854	.146	.130	.103
EOC2	.104	-.018	.031	.043	.115	.186	.094	.749	.130	.036	.027
EOC3	.025	.052	-.014	-.014	.011	.209	-.073	.834	.188	.183	.140
EOC4	.119	.051	.010	-.040	.078	.259	-.078	.786	.142	.249	.069
EMC1	.039	.012	.021	.008	.017	.248	.002	.207	.128	.886	.029
EMC2	.053	.010	-.016	-.023	.024	.284	.045	.151	.131	.896	.074
EMC3	.040	.014	.054	-.048	.040	.208	.032	.165	.096	.875	.051
PQ	.068	.048	.086	.091	.076	.098	.102	.143	.811	.094	.073
CE	.132	.080	.009	.098	.112	.153	.156	.157	.770	.087	.054
TL	.096	.169	.035	.123	.152	.179	.058	.129	.749	.117	.094
US	.115	.159	.059	.085	.078	.081	.046	.146	.829	.069	.044

Extraction Method: Principal Component Analysis

Rotation Method: Varimax with Kaiser Normalization

Rotation converged in 7 iterations

CFA was used to establish the convergent/discriminant validity and construct reliability (CR). Convergent validity is assessed through criterion of Average Variance Extracted (AVE) by each construct should be > 0.5 or 50% [84]. Discriminant Validity is established through two criteria: (1) Square root of Average Variance Extracted (AVE) is greater than inter construct correlation i.e. √AVE > γ or AVE > γ2 (2) Maximum Shared Variance (MSV) is less than Average Variance Extracted (AVE) i.e. MSV < AVE [85]. A construct is considered reliable if its Composite Reliability (CR) > 0.7 [85].

CFA using AMOS-20 with Maximum Likelihood (ML) Approach is performed for all the *first order constructs*. The model fits well with fit statistics, *χ*2/d*f* = 1.476, CFI = 0.97, PNFI = 0.80, PGFI = 0.697, RMSEA = 0.045, RMR = .028. Results of convergent and discriminant validity along with construct reliability of first order constructs are presented in Table 3. Three basic assumptions of multivariate analysis i.e. normality, linearity, and homoscedasticity are examined for all the constructs, no statistically significant violations are observed.

**Table 3:** Convergent & discriminant validity and construct reliability results of first order constructs.

**Construct**	**CR**	**AVE**	**MSV**	**EMC**	**TMC**	**QCU**	**IM**	**PM**	**CI**	**KSA**	**QAC**	**EOC**	**OE**	**SVP**
**Criteria**	**> 0.7**	**> 0.5**	**MSV< AVE**	**√AVE > γ**										
**EMC**	0.945	0.852	0.282	**0.923**										
**TMC**	0.977	0.913	0.250	0.145	**0.956**									
**QCU**	0.953	0.835	0.283	0.088	0.432	**0.914**								
**IM**	0.969	0.887	0.279	0.035	0.224	0.213	**0.942**							
**PM**	0.966	0.878	0.279	-0.009	0.189	0.227	0.528	**0.937**						
**CI**	0.970	0.890	0.272	0.100	0.305	0.257	0.493	0.481	**0.943**					
**KSA**	0.947	0.818	0.327	0.531	0.131	0.187	0.007	0.022	0.109	**0.904**				
**QAC**	0.949	0.823	0.272	0.075	0.337	0.222	0.499	0.479	0.522	0.040	**0.907**			
**EOC**	0.904	0.706	0.327	0.448	0.224	0.147	0.003	-0.004	0.130	0.572	-0.047	**0.840**		
**OE**	0.877	0.640	0.183	0.329	0.343	0.368	0.209	0.309	0.355	0.371	0.307	0.428	**0.800**	
**SVP**	0.950	0.864	0.283	0.201	0.500	0.532	0.145	0.095	0.169	0.228	0.091	0.298	0.294	**0.929**

**γ = Inter construct correlation**

**(SQRT-AVE) Square Root of Average Variance Extracted is on the diagonal**

QMS-Top Management practices, QMS-Core practices and HPWS practices are conceived as second order factors. Transforming first order factors into second order is consistent with the literature and a normal practice in organizational research. At step one, eleven composite measures or summated scale are made by combining several individual items into a single composite measure. At step two, three second order factors are formed from these composite measures (1) QMS-Top Management Practices (QMS-TMP) consist of three composite measures or summated scales: Top Management Commitment(TMC), Strategic Vision and Planning(SVP) and Quality Culture(QCU) (2) QMS-Core Practices (QMS-CP) consist of four composite measures or summated scales: Process Management (PM), Customer Focus (CF), Supplier Management (SM) and Continuous Improvement (CI) and (3) High Performance Work System consists of three composite measures or summated scales: Employees Knowledge Skills and Abilities (KSA), Employees Opportunities to Contribute (EOC) and Employees Motivation and Commitment (EMC) whereas Organizational Effectiveness remains as a first order construct and a composite measure with four items: Product Quality (PQ), Cost Effectiveness (CE), Timeliness (TL) and User Satisfaction(US). CFA of complete measurement model is conducted to examine the reliability and convergence/discriminant validity of second order constructs. All the first order constructs evidently converge on relevant second order factors. Results of convergent and discriminant validity along with construct reliability of second order constructs are shown in Table 4.

**Table 4:** Convergent & discriminant validity and construct reliability results of second order construct.

**Construct**	**CR**	**AVE**	**MSV**	**QMS-TMP**	**QMS-QLP**	**HPWS**
**Criteria**	**> 0.7**	**> 0.5**	**MSV< AVE**	**√AVE > γ**		
**QMS-TMP**	0.753	0.504	0.171	**0.710**		
**QMS-CP**	0.800	0.500	0.171	0.413	**0.707**	
**HPWS**	0.767	0.525	0.118	0.343	0.076	**0.725**

**γ = Inter construct correlation**

**(SQRT-AVE) Square Root of Average Variance Extracted is on the diagonal**

## 5. Test results of structural model

Byrne [86] stated that *“Structural equation modeling is a statistical methodology that takes a confirmatory (i*.*e*., *hypothesis-testing) approach to the analysis of a structural theory”*. Structural equation modeling represents a series of causal relationships generating observations among multiple variables simultaneously and is preferred over other multivariate techniques like correlation and regression [86]. SEM using AMOS 20 is used to empirically determine the fit of the proposed model. Results of measurement model and structural model are illustrated in Table 5. Considering the suggested values of fit indices, an examination of the goodness-of-fit indices shows a good fit of the model to the data.

**Table 5:** Global model fit of the measurement model and structural model.

Goodness-of-fit statistics	Measurement Model	Structural Model	Model fit Criteria
***χ*2/d*f***	**1265.243/804 = 1.574**	**1177.529/802 = 1.468**	**< 3.00**^a^
**CFI**	**0.962**	**0.969**	**> 0.95**^b^
**PNFI**	**0.843**	**0.847**	**> 0.5**^b^
**PGFI**	**0.720**	**0.727**	**> 0.5**^b^
**RMR**	**0.044**	**0.042**	**< 0.05**^b^
**RMSEA**	**0.049**	**0.044**	**< 0.08**^b^
**CAIC (Default model)**	**1906.826**	**1832.073**	***<* Saturated model and independence model**^b^
**CAIC (Saturated model)**	**5852.017**	**5852.017**	
**CAIC (Independence model)**	**13295.847**	**13295.847**	

^a^Hair et al. [85],

^b^Byrne [86]

Table 6 shows SEM results of direct, indirect and interaction relationships including the related hypotheses, standardized parameter estimates, standard errors and C.R values of the proposed relationships. As shown in the table, all hypotheses are supported.

**Table 6:** Construct structural model.

**Direct Links in the model**					
	Hypotheses	Standardized parameter estimates			Results
		Estimate	S.E	C.R	
QMS-TMP ⟶ OE	H1	0.185**	0.095	2.111	Supported
QMS-TMP ⟶ QMS-CP	H2	0.409***	0.084	4.408	Supported
QMS-TMP ⟶ HPWS	H3	0.338***	0.083	3.728	Supported
QMS-CP ⟶ OE	H4	0.182**	0.095	2.310	Supported
HPWS ⟶ OE	H6	0.321***	0.097	3.956	Supported
**Indirect Links in the model** §					
QMS-TMP → QMS-CP → OE	H5	0.081**	0.049	1.653	Supported (Partial mediation)
QMS-TMP → HPWS → OE	H7	0.118***	0.055	2.145	Supported (Partial mediation)
**Interaction Link in the model**					
QMS-CP x HPWS → OE	H8	0.331***	0.037	8.94	Supported

***P < 0.01,

**P < 0.05

^§^ For indirect links in the model:

Bootstrap samples- 2000; Bias corrected

Confidence Interval (CI) - 95%

## 6. Discussion and implications

The main objective of this research work was to develop an integrated framework of QMS and HPWS to investigate the relationships among QMS and HPWS practices and analyse the conjoint implementation of QMS and HPWS on organizational effectiveness. The objective was accomplished through the empirical analysis of the hypothesized structural model, established through a comprehensive literature review. A system perspective was assumed and the proposed model was validated using concept of fit. The outcomes of the study suggest that QMS-Top Management practices are enablers or keys, which drive both QMS-Core and HPWS practices to achieve organizational effectiveness. Simultaneous implementation of QMS and HPWS strengthen each other and contributes to achieve organizational effectiveness. To begin with, as hypothesis **(H1)** indicates that there is a significant positive relationship between QMS-Top Management practices and organizational effectiveness. This finding is consistent with the results by Ahire and O’Shaughnessy [54], Saleh and Sweis [87], Jimoh et al. [14] and Abbas [13]. QMS is inherently integrative and a strategic level priority. Top management with a quality oriented strategy, boosts the spread of quality philosophy through the organization, hence enhances its effectiveness at all levels. TSOs can achieve consistent and lasting excellence if their top management is able to integrate quality into organizational strategy. Hypothesis **(H2)** shows a significant positive relationship between QMS- Top Management Practices and QMS- Core Practices. As stated by Murphy and Leonard [82], when viewed as a strategic imperative, QMS top level acts as a driving force for its core elements. The finding is further strengthen by Tahir et al. [63] who argued that organizational culture consisting of top management commitment and common internal/external infrastructure are antecedent to enable QMS core practices. Result of hypothesis **(H3)** depicts that QMS-Top Management Practices have a significant and positive impact on HPWS practices. It means if top management is determined to bring about valued organisational outcomes, it needs to design and implement internally consistent policies aimed at enhancing knowledge, skills and abilities of human capital, mutual collaboration, empowerment and devotion. Hypothesis **(H4)** shows that QMS-Core Practices have a significant and positive impact on organizational effectiveness. This result is in conjunction with the outcomes of many other studies where QMS is either considered as a first order construct [9, 10, 35, 36, 11], or a multidimensional second order construct [12, 13, 14, 33, 34, 4, 15] while establishing its relationship with organizational effectiveness. Hypothesis **(H5)** indicates that QMS-Core practices have a partial mediating effect on the relationship between QMS-Top Management practices and organizational effectiveness. This finding identifies the direct and indirect effects of QMS practices on organizational effectiveness at two distinct levels and describes how interrelationships among QM components create synergistic benefits for the organization. QMS Core practices act as contributing factor, providing necessary support to the relationship between QMS Top Management practices and organizational effectiveness. Hypothesis **(H6)** shows a significant positive link between HPWS practices and organizational effectiveness. This outcome supports the configurational approach [6, 7, 8, 42] which emphasizes that bundles of internally consistant HR practices may universally exert positive effects on organizational outcomes. This result is also in line with the Resource Based View (RBV), that advocates the significance of combined resources to achieve organizational effectiveness [88]. Result of hypothesis **(H7)** shows that QMS-Top Management Practices have a positive impact on organizational effectiveness indirectly through HPWS practices. This finding supports the belief that top management task is to foster and sustain a shared organizational culture where the main activities of the organization are not ‘*managed*’ by the top managers in the sense of being directed and controlled by them. They need to create a culture of constructive discourse where employees are considered as valuable assets and key players, if effectively managed through training and development, motivation and empowerment, capable to contribute significantly to organizational effectiveness. Lastly, the result of hypothesis **(H8)** shows that the interaction between HPWS practices and QMS-Core practices has a significant positive impact on organizational effectiveness. The result is in line with the finding of Alkhazali et al. [22]. True QMS can only be brought about by recognition of the value of interactive professionalism and complementary diversity. It means neither QMS-Core practices nor HPWS practices alone are enough to achieve maximum organizational effectiveness. When an organization is able to create favorable conditions with respect to employees and core management practices, a positive effect on organizational effectiveness will occur and it will be more than the sum of the individual effects. Upon reflection, this result makes good intuitive sense. Skilled, motivated and empowered employees with superficial and weak QMS-Core practices in play, will not make much of a contribution to the organization. Likewise, sound and well established QMS-Core practices with the unskilled, unmotivated or suppressed workforce will also fall short to achieve maximum performance.

The exploration of relationship between QMS practices (at both levels) and HPWS in a conjoint implementation is inspiring for practitioners. Scholars, involved in development of integrated framework of QMS and HPWS, should concentrate on the multidimensionality of the QMS construct at different management levels as the nature of relationship of QMS and HPWS is different at different management levels. Managers can utilize this model periodically to ascertain the effectiveness of their organization in the QM journey.

### 6.1 Research limitations

All possible efforts were made at the design stage of this study to achieve reliable and valid findings as shown in section 4. Nevertheless, this study has numerous limitations. The study is limited in scope to the TSOs of Pakistan and limited in time as it provides a cross sectional view of the proposed theory. Moreover, this research work did not include financial measures of performance. The moderating effects of contextual factors (firm size, firm type and ISO-9000 registration) on the proposed model could not be examined as well. However, with the above limitations acknowledged, the study provides a conceptually insightful and empirically validated framework for a conjoint implementation of QMS and HPWS in the organization.

### 6.2 Directions for further research

The empirically validated strategic model presented in this study can be helpful for researchers to further refine the 7framework and investigate its suitability in services and manufacturing industry. It is further recommended to incorporate the Resource Management practices (e.g. capacity utilization of space, capacity utilization of equipment and capacity utilization of workforce etc.) in the model since it is assumed that these practices build the supportive base of both QMS and HPWS. The contextual factors should also be catered for in future studies.

## 7. Conclusion

This paper aims at a better understanding of the interplay of QMS and HPWS practices and their direct and indirect impact on several criteria of organizational effectiveness. It develops an integrated framework of QMS and HPWS to explore the conjoint implementation of both on organizational effectiveness. The known QMS practices are classified and analysed at two distinct levels: Top Management practices and Core practices. The rationale here is that, there is a significant relationship between top and core level which needs to be focused on for a successful QMS implementation. QMS Top Management practices have proven to be a driving force for QMS Core and HPWS practices. It means that lack of quality culture, effective planning and top management commitment will have a negative effect on the implementation of QMS and HPWS. QMS-Core practices and HPWS practices are influenced by QMS Top Management practices and also act as a mediator between QMS Top Management practices and organizational effectiveness. With regards to human resources, this study elucidates that a successful implementation of HPWS drives an organization through workforce involvement, dedication and empowerment, instead of employee control. In any organization where HPWS is well placed, employees realize their responsibility towards attainment of success; they are more knowledgeable, more hardworking and add more to organization. They have authority, information, awareness and opportunity to perform at the top level. Finally, this study also contributes by empirically analysing the interaction effect of QMS-Core and HPWS practices on organizational effectiveness which shows that neither QMS-Core practices nor HPWS practices alone are enough to achieve optimum organizational effectiveness.

## Supporting information

S1 Appendix Measurement scales, survey items, and their sources. (DOCX)
