# Supplementary material for: Do they really coexist? An empirical analysis of a conjoint implementation of Quality Management System and High Performance Work System on organizational effectiveness
Source: PLoS One. 2020 Mar 9;15(3):e0229508. doi: 10.1371/journal.pone.0229508 (PMC7062248; doi:10.1371/journal.pone.0229508)
Supplement: S1 Appendix — (DOCX) [file pone.0229508.s001.docx]

**Appendix A**

| **Top Management Commitment**  Adapted from Ahire et al.(1996) and Cua et al. (2001) | | |
| --- | --- | --- |
| TMC1 | Our quality goals are clearly defined by top-management | |
| TMC2 | Top management is personally responsible to ensure quality products and quality enhancement | |
| TMC3 | All key department heads in our organization accept their responsibility for quality | |
| TMC4 | Top management provides sufficient resources in the efforts of quality enhancement | |
| **Strategic Vision & Planning**  Adapted from Cua et al. (2001) | | |
| SVP1 | Our planning process is very comprehensive and well-structured which frequently sets and evaluates short and long-term goals | |
| SVP2 | Development and execution of our organizational strategies and plans are established through data regarding customer’s needs and organization’s capabilities. | |
| SVP3 | Our organization always strives for achievement of ‘Best Practices’ | |
| **Quality Culture**  Adapted from Terziovski et al.(2003) | | |
| QCU1 | Top Management has allocated the responsibility for quality down to all organizational members | |
| QCU2 | Quality planning is an essential fragment of the strategic plan | |
| QCU3 | Quality systems and procedures are clearly understood throughout the organization | |
| QCU4 | Benchmarking is comprehensively utilized to enhance products quality and processes | |
| **Process Management**  Adapted from Tarı et al. (2007) | | |
| PM1 | We ensure continuous control and enhancement of key processes | |
| PM2 | Every effort is made to avert faulty products/services | |
| PM3 | Quality measures are the essential part of all the processes | |
| PM4 | All the employees are capable to evaluate their related processes | |
| **Customer Focus**  Adapted from Samson et al. (1999) | | |
| CF1 | We are well aware of our customers present and future needs | |
| CF2 | Customer requirements are properly circulated to and fully understood by all the employees | |
| CF3 | Customer’s satisfaction is systematically and repeatedly examined | |
| CF4 | Customer complaints/feedback are utilized as an essential source to bring improvements in on-going processes | |
| **Quality Assurance and Control**  Adapted from Saraph et al. (1989) | | |
| QAC1 | We have an autonomous Quality Assurance & Control department in our organization | |
| QAC2 | The product is thoroughly tested for quality by QAC department before they are handed over to the customer | |
| QAC3 | There is a high degree of coordination between QAC and other departments | |
| QAC4 | There is a strong interaction between QAC department and top management | |
| **Continuous Improvement**  Adapted from Rungtusanatham et al. (1998), Tarı et al. (2007) | | |
| CI1 | Everyone in the organization is responsible for quality improvement | |
| CI2 | Continuous improvement of quality is ensured in every work process all over the organization | |
| CI3 | All employees evaluate their tasks to identify the areas of improvement | |
| CI4 | Special measures are taken to determine loss of time and cost in all critical processes | |
| **Employees Knowledge, Skills and Abilities**  Adapted from Saraph et al. (1989), Bello-Pintado, (2015) | | |
| KSA1 | We utilize multiple selection methods throughout the staffing procedure (e.g. interviews, personality and aptitude tests, etc.) | |
| KSA2 | We have adequate resources for better education and training of our employee | |
| KSA3 | Our employees are thoroughly trained for specific work-skills | |
| KSA4 | Employees who have been in the organization for a longer duration, have learnt to perform more tasks | |
| **Employees Motivation and Commitment**  Adapted from Bayo-Moriones and Galdon-Sanchez (2010), Bello-Pintado, (2015) | | |
| EMC1 | Employees are recognized and rewarded for their performance through a clear and effective appraisal system | |
| EMC2 | Our incentive system is completely impartial at rewarding employees who achieve organizational objectives | |
| EMC3 | Our organization is highly committed to maintain permanent contract with our employees to give them job security. | |
| **Employees Opportunity to Contribute**  Adapted from Michaelis et al.2015, Bello-Pintado, (2015) | | |
| EOC1 | Employees have full opportunities to suggest improvements in the processes and procedures | |
| EOC2 | Employees are always invited to attend scheduled meetings where they identify, evaluate, discuss and offer solutions regarding work-related issues | |
| EOC3 | Organizational performance is formally communicated to employees on regular basis | |
| EOC4 | The employees are empowered to decide when, how and in which order tasks have to be carried out | |
| **Organizational Effectiveness**  Adapted from Cua et al. 2001, Kristensen et al.2010 | | |
| **Product Quality (PQ)** | | Product’s conformance to Specification & Standard |
| **Cost Effectiveness (CE)** | | Unit cost of production |
| **Timeliness (TL)** | | Organization’s On-Time delivery performance |
| **User Satisfaction (US)** | | Level of user’s satisfaction known through feedback |
